# "Maybe this is just not the place for me:" Gender harassment and discrimination in the geosciences

**DOI:** 10.1371/journal.pone.0268562

**Published:** 2022-05-18

**Authors:** Allison Mattheis, Erika Marín-Spiotta, Sunita Nandihalli, Blair Schneider, Rebecca T. Barnes

**Affiliations:** 1 California State University, Los Angeles, California, United States of America; 2 University of Wisconsin-Madison, Madison, WI, United States of America; 3 University of Kansas, Lawrence, KS, United States of America; 4 Colorado College, Colorado Springs, CO, United States of America; University of Alberta, CANADA

## Abstract

Rampant gender-based harassment and discrimination are recognized problems that negatively impact efforts to diversify science, technology, engineering and mathematics (STEM) fields. We explored the particularities of this phenomenon in the geosciences, via focus groups conducted at STEM professional society meetings, with the goal of informing interventions specific to the discipline. Using grounded theory analysis, two primary drivers for the persistence and perpetuation of gender-based harassment in the geosciences were identified: a particular history of power dynamics and maintenance of dominant stereotypes, and a pattern of ineffective responses to incidents of harassment and discrimination. Informed by intersectional feminist scholarship by women of color that illustrates how efforts to address the underrepresentation of women in STEM without attending to the overlapping impacts of racism, colonialism, ableism, and classism will not succeed, we view harassment and discrimination as structural problems that require collective solutions. Continuing to recruit individuals into a discipline without changing its fundamental nature can tokenize and isolate them or encourage assimilation and acceptance of deep-seated traditions no matter how damaging. It is the responsibility of those in power, and especially those who hold more privileged status due to their social identities, to contribute to the dismantling of current structures that reinforce inequity. By providing explanatory illustrative examples drawn from first-person accounts we aim to humanize the numbers reported in workplace climate surveys, address gaps in knowledge specific to the geosciences, and identify interventions aligned with an intersectional framework that aim to disrupt discriminatory practices endemic to the geosciences and larger STEM community.

## Introduction

A landmark report released in 2018 by the National Academies of Science, Engineering and Medicine found that sexual harassment and related discriminatory behaviors are fostered in strongly hierarchical environments with severe power imbalances, such as occur in academia [1]. These conditions are particularly salient to the geosciences, a broad discipline that encompasses the earth (including atmospheric and ocean sciences) and space sciences with large gender and racial disparities in representation from the student to more senior level positions in academia and industry. Persistent low diversity in the geosciences [2] maintains existing hierarchies and increases vulnerability of historically excluded groups to social isolation and to both identity-based and more general exclusionary behaviors; these circumstances deny opportunities for career advancement to large groups of people in our society. Promising geoscientists can be driven out of the field both through bias that continues to reward those with majority identities, and hostile behaviors that target individuals early in their careers [3–5].

Rampant harassment and discrimination are recognized problems in STEM fields [6, 7] and counter efforts to increase diversity, equity and inclusion in the profession [1, 8]. Through a qualitative, grounded theory approach we explored the particularities of this phenomenon in the geosciences, and found that persistent dominant stereotypes and power structures, along with a history of ineffective responses to incidents of harassment and abuse perpetuate these behaviors in the discipline. By providing explanatory illustrative examples drawn from first-person accounts and offering suggestions for interventions aligned with an intersectional framework we aim to disrupt pervasive discriminatory practices within our field.

### Study context: Persistent low gender and racial diversity in the geosciences

The underrepresentation of white women and people of color of all genders is especially noticeable in the geosciences. Women earn approximately 40% of all undergraduate degrees awarded in the earth, atmospheric, and ocean sciences [9, 10], yet make up less than 30% of geoscience faculty [11–13]. Many geoscience departments may have only two or three (white) women faculty and no faculty of color. Representation decreases at advanced career stages [11], reflecting failures of efforts designed to address underrepresentation as a "pipeline problem" [14]. The geosciences also have the smallest representation of historically excluded groups [15]; Black, Hispanic, American Indian, Alaska Native, and Asian Pacific Islander women together represent only 5% of bachelor’s degrees and 7% of tenure-track faculty [9]. The number of PhDs awarded to these groups has seen little to no increases in the last 40 years, according to an analysis of the earth, atmospheric and ocean sciences subfield data from the 2016 Survey of Earned Doctorates [2]. Women of color are especially underrepresented, with only 1.46% of doctorates in geoscience subdisciplines awarded to Native American, Black or Hispanic women [2]. These low numbers can lead to isolation and increased vulnerability to sexual and racial harassment [16–18]. International scholars and those who identify as transgender, genderqueer, or gender non-conforming are also at greater risk of being targeted by exclusionary behaviors [19–22]. Recent attention in the geosciences has focused on unique challenges posed by expectations of remote fieldwork, required for many undergraduate geology programs, and which can create unsafe conditions for those who do not conform to the stereotype of geoscientists as white, able-bodied, cisgender, straight men [23–25]. The geosciences address issues of key importance to societies around the globe, especially in the face of ongoing climate change; it is essential that this community reflect the needs and input of a much broader range of people [26]. Ultimately, making sure anyone who wants to pursue a career in the geosciences is able to do so free of harassment and discrimination is imperative.

### Literature review

This study engages with two broad areas of relevant extant research: work that has investigated sexual harassment and gendered discrimination in STEM fields, and studies of how organizational and institutional climates and practices relate to these behaviors.

### Prevalence of sexual harassment and gendered discrimination in academic STEM workplaces

Sexual harassment is an expression of institutionalized sexism, resulting from unequal workplace gender relations [27]. Sexual harassment is conceptualized as a range of behaviors, including gender harassment, unwanted sexual attention, and sexual coercion [28–30]. Research demonstrates that most harassment in male-dominated workplaces is more accurately characterized as gender-based [31], and implicit bias, microaggressions, and other unprofessional behaviors and attitudes are associated with hostile sexist experiences [8, 32, 33]. Scholars have debated how to frame sexual harassment as an issue of women’s rights, of workplace safety, or as a civil rights problem [27]. The entry of women into traditionally male-dominated workplaces led to the recognition of sexual harassment as a societal problem. Due to its effect on women leaving their jobs, sexual harassment is also an economic problem; when sexual harassment contributes to women leaving STEM altogether, it becomes a science problem [34].

The hierarchical and traditional nature of universities and other academic workplaces create conditions in which sexual harassment can persist [1]. As part of its efforts to collect national data on sexual assault and sexual misconduct in higher education, the Association of American Universities surveyed more than 180,000 students in 2019 about their experiences [19]. Among all students, 41.8% reported experiencing at least one sexually harassing behavior since enrollment; undergraduate transgender, non-binary, genderqueer or questioning and (cisgender) women students reported experiencing the highest rates of sexual misconduct not classified as assault, and graduate and professional students were the most likely to be subject to sexually harassing behavior by a faculty member or instructor [19]. A survey of experiences in the 1990s found that 20 to 50% of women faculty, staff, and administrators had experienced sexual harassment in their academic workplaces [35]; these statistics have changed little in the past few decades [1]. Persistent gender inequities continue to cause challenges for women in higher education workplaces [36] and serial faculty harassers are often passed along to new institutions rather than pushed out of the field altogether [37].

The geosciences are not immune to these behaviors. Research reveals the prevalence of sexual harassment experienced by trainees in disciplines that involve field training [38] and how sexual and racial harassment disproportionately affect women of color’s access to research and educational opportunities in the planetary sciences [3]. Fifty one percent of respondents to a survey of members of the Earth Science Women’s Network (ESWN) reported experienced sexual harassment or assault within their work environment [39]. Vulnerability increases during travel to conferences and during training and research at off-campus field sites, away from regular support networks [39]. Seventy one percent of women received inappropriate comments and 26% reported experiencing sexual assault in a survey of field scientists [40]. Women students disproportionately reported unwanted sexual attention from their superiors, often faculty or graduate students. Few were aware of how to report sexual misconduct. This problem is urgent, as a majority of U.S. geology programs require field training and field camp attendance continues to increase [41–43].

### Impacts of harassment and discrimination in the workplace

Aspects of institutional climates associated with greater prevalence of sexual harassment include tolerance of associated behaviors, gendered perceptions about certain professions, and lack of overall diversity [28, 44]. In the geosciences, stereotypes of geoscientists as able-bodied, cisgender, white men alienate those who do not conform [45, 46], and can contribute to field safety concerns [24, 47]. High-profile cases have raised public awareness about a permissive culture of harassment and bullying in academic STEM [48–50] and the National Academies of Sciences expelled its first member for violating their sexual misconduct policy [51]. Multiple studies have demonstrated that women are disproportionately affected by negative workplace environments or climate, defined by a set of formal and informal procedures, practices, and policies [52–55]. Some argue that all forms of harassment, including that based on gender, are motivated by a desire to preserve social status [56].

Organizational climates can be conceptualized as comprised of three primary components: affective (social interactions and interpersonal relationships), instrumental (work processes, structures and extrinsic rewards), and cognitive (autonomy, innovation, and intrinsic rewards) [57]. Harassment and discrimination can cause damage to all three of these aspects of a workplace—trusting relationships are broken not only by offenders but also by those who fail to act in support of targets, access to necessary resources or advancement opportunities can be restricted by harassers, and retaliation for rejecting advances or reporting harassment can harm individuals and team functions. Hostile environments can lead to long-term emotional and health risks akin to trauma [16, 58].

Some well-meaning approaches to address these negative impacts can unintentionally reinforce the existing organizational structures that allow for harassment and discrimination to occur. The ostensible protections provided by federal legislation have been limited; as summarized in the NASEM report, "symbolic compliance with Title IX and Title VII has resulted in policies and procedures that protect the liability of the institution but are not effective in preventing sexual harassment" [1] p. 4. Higher education is second only to the military in reports of sexual harassment, and this problem is directly related to other forms of racial and gendered disrespect [59]. A multi-level approach to transform workplace climate is needed [60]: at the institutional level, by addressing academic cultures; structurally, through policies and processes that guide professional conduct and response to sexual harassment; and individually, through education and empowerment of all genders [60, 61]. Because of the links between harassment based on gender, race, and other minoritized identities in STEM workplaces, the 2018 NASEM report included as its first recommendation "Create diverse, inclusive, and respectful environments" [1].

### Conceptual framework: Intersectionality and disciplinary norms in STEM fields

In order to address problems of bias and underrepresentation, it is important to understand the forces that contribute to these current circumstances. Thus, the ADVANCEGeo project, and associated data collection, is guided by a conceptual framework that aims to apply an intersectional understanding of social structures and resulting oppression to understand how exclusion occurs in the geosciences [47, 61].

### Intersectionality

The term "intersectionality" was first coined by legal scholar Kimberlé Crenshaw in two foundational articles [62, 63] that applied Black feminist and Critical Race Theory analyses to demonstrate how legal structures did not account for the overlapping oppressions of sexism and racism faced by Black women in the workforce. Multiple disciplines have adopted intersectionality as an analytic and interpretive framework with the purpose of engaging a range of issues surrounding social justice and power dynamics and how systems of oppression disproportionately affect already underserved groups [64]. Intersectionality serves as a tool to examine how individual and collective disadvantage are impacted by multiple axes of power and inequality [65]. Intersectionality also illustrates how aspects of identity, especially race and gender, while explicit within the U.S. legal and political systems, are not unitary, mutually exclusive identities. Rather, all aspects of one’s identity reinforce (and in some cases counteract) complex social inequalities [66]. Further, this narrow construction of identities (specifically race and gender) within the legal system invites resistance, further marginalizing those most negatively impacted [62, 63]. Intersectionality as an analytic lens is connected to the activist efforts of women of color and academic applications should acknowledge these roots in social movements advocating for systemic change [66].

In order to best identify how and which structures within the geosciences reinforce and maintain existing power dynamics, we use an intersectional lens, drawing on scholars’ [65–68] proposals of how intersectionality as an analytic tool can be used to interrogate organizational structures and the overlap of oppressive hegemonic influences such as racism, sexism, heteronormativity, ableism, and classism beyond investigations of specific identity categories. At the same time, we acknowledge and highlight the importance of white supremacy and western heteropatriarchy as social forces that shape the contemporary context of the geosciences in the U.S., and positions those with minoritized identities in terms of race, ethnicity, gender, sexuality, and ability at additional risk for harassment and discrimination.

### Harassment and discrimination as institutional problems

Using an intersectional lens we reframe harassment and discrimination as problems reflecting broader cultural practices within the geosciences, an understanding illustrated in the 2018 NASEM report. Specifically, academic environments in science, technology, engineering, mathematics and medicine are identified as traditionally male-dominated and characterized by rigid hierarchies that disproportionately empower senior members of these communities and disempower trainees and supervisees [1]. Further, women of color, sexual and gender minority people, disabled people, and people who have migrated or immigrated to the U.S. are at greater risk of experiencing harassment because existing white supremacist power dynamics place them in a subordinate position [1]. Assumptions of gender and race are reinforced through practices and beliefs in STEM fields, resulting in women and people of color perceiving their workplaces as less supportive and equitable than white male colleagues [14, 69, 70]. Importantly, the biases and stereotypes that contribute to these conditions are not only upheld by those in power or with dominant identities [71]; normative influences operate hegemonically, and can socialize even those with marginalized identities into cultural practices that reinforce rather than deconstruct discrimination in STEM fields. Confronting harassment therefore requires intervention efforts that go beyond efforts to change individual beliefs and behaviors.

## Methods

This exploratory investigation served as the first stage of a larger effort to address harassment and discrimination in the geosciences. Using a qualitative inquiry approach allows for open exploration of this phenomenon from multiple perspectives, recognizing that people make sense of social experiences via their observations, that individuals often iteratively seek to find meaning [72]. Specifically, we used grounded theory as a mode of qualitative inquiry because it is particularly well-suited to social justice studies that seek to inform practices and policies [73]. In order to gather knowledge that would allow us to develop tailored interventions as part of our program, we were guided by a general exploratory research question: *How have harassment and discrimination been experienced by members of the geosciences community*? Following framing of qualitative research as learning [74], we aimed to create safe and supportive spaces where participants could share their experiences honestly and contribute to our overall understanding. Based on our broader goal of reducing bias and discrimination based on gender, race, and other aspects of social identity, our study design was informed by transformative epistemologies with the explicit goals of promoting social change; our interpretation of the individual experiences of participants was therefore connected to our broader understandings of social structures and oppressions [73]. Our ethical commitments to participants were central to our engagement with this research, and data collection and analysis decisions were guided by the intersectional philosophy described above. This research was approved by California State University Los Angeles (#1071740–1) and University of Wisconsin Madison (#2017-0630-CP003) Institutional Review Boards, and all participants provided oral consent to participate.

### Data collection and analysis

Focus groups are a mode of qualitative data collection that gather participants who have similar experiences or characteristics to provide their individual perspectives about an issue of shared interest [75]. In a focus group interview, a facilitator guides a small group of people through a series of questions that are purposefully sequenced to elicit perspectives, feelings, and thinking about a topic, while allowing space for participants to direct the conversation in particular ways [75]. We conducted five focus group interviews at the national meetings of three professional societies—one that was geoscience specific and two with a broader STEM focus but attended by many geoscientists with interdisciplinary interests. Participants were recruited online through social media accounts and listservs associated with groups including the Earth Science Women’s Network and Association for Women Geoscientists and on-site at the meetings through flyers. The five focus groups ranged in size from 2–11 participants, and all participants provided consent for their conversation to be used for research purposes prior to the start of each interview in accordance with IRB guidelines. In order to built trust and rapport among participants and with the researchers, facilitators read an introductory script that emphasized the need to respect confidentiality and reiterated our learning goals and intended use of their input; participants were also asked to skip any questions they did not feel comfortable answering and invited to ask clarifying questions whenever necessary. We also provided a handout with a list of resources for survivors of sexual assault or harassment in the event that sharing past experiences of violence may have been a triggering event and encouraged participants to contact researchers if they had follow-up comments or questions, or if they wished for us to remove their responses from the data set. All focus groups were one hour in length and were audio-recorded and transcribed verbatim for accuracy. All data were collected between October 2017 and February 2018.

Our analysis aimed to develop an explanatory model in line with transformative grounded theory practice [73]—in this case, we looked to identify how discrimination and harassment are experienced in ways that are specific to the geosciences as a broad disciplinary field, and what aspects of the field contribute to these experiences. Our analytic approach was reflexive and iterative, but systematic and guided by the overall purpose of our study [72]. Shortly after all focus groups were completed, [AUTHOR #3] conducted a preliminary analysis of all five transcripts by reading through each one and preparing a memo outlining three primary themes she identified; these were shared and discussed with the larger group of investigators. Before continuing our analysis, we expanded our review of the extant literature to further inform our understanding of the overall phenomenon. Next, [AUTHOR 1] used the qualitative software analysis program Dedoose [76] to complete a first round of line-by-line coding of the five focus group transcripts, using an inductive coding approach to identify themes in the overall data set [77]. She also wrote researcher memos throughout the coding process to document emerging interpretations and identify questions for the other authors. A summary of [AUTHOR 1]’s preliminary analysis, including a list of codes and sample excerpts, was then shared with [AUTHORS #2, 4, and 5]. Following a structured process, they prepared individual memos that identified connections between existing codes in order to begin collapsing these into broader explanatory categories. [AUTHOR 1] then consolidated these memos and used these additional analyses to identify primary findings, which were then again reviewed, revised, and confirmed by [AUTHORS #2, 4, and 5]. Our goal was not to focus on descriptive analysis, but rather to "reach across individuals and events to reveal a collective analytic story" [73]. Trustworthiness in this study was ensured through the collaborative development of a protocol to guide each focus group, the presence of at least two researchers at each focus group to collect additional observation notes during data collection, and an iterative process of analysis that allowed for reflexivity and consideration of multiple possible explanations and perspectives. These strategies allowed for triangulation via methods, data sources, investigators, and additional insight into research findings based on theory [78]. As recruitment materials identified the focus of our project on the geosciences, most participants were geoscientists, but a few joined focus groups to discuss their experiences in other STEM fields. These data were included in our analysis to inform our understanding of how harassment and discrimination operate uniquely and similarly across fields, but the specific examples included in this manuscript are from participants who identified themselves as geoscientists.

### Researcher positionality

In keeping with our research approach, we reject claims of complete objectivity but acknowledge the need to address individual biases based on different social locations. [AUTHOR #1] is an Associate Professor of Education at a regional public university who is a white cisgender queer woman. [AUTHOR #3], a biracial gay woman, was a graduate research assistant at the time of data collection and now works as a counselor. [AUTHOR #2] is Professor of Geography at a primarily white land-grant institution in the Midwest and is a white Hispanic cisgender heterosexual woman [AUTHOR #5] is an Associate Professor of Environmental Science at a small liberal arts school who is a white cisgender queer woman. [AUTHOR #4] is a white cisgender bisexual woman, who was a postdoctoral researcher at the time of data collection and is now an Associate Researcher for a state geological survey. Our involvement in the research process varied based on attendance at the different conferences and participation in data collection and analysis procedures. [AUTHORS 1, 2, and 3] facilitated the focus groups either individually or in pairs, and [AUTHORS 4 and 5] were present as note-takers for 3 focus groups. As a collective endeavor, we aim to include multiple perspectives in order to understand a complex phenomenon.

### Participants

A total of 23 individuals participated in the five focus group interviews. Of these participants, 83% were women, 26% were people of color, and 17% identified as LGBTQ+. A range of experience was reflected, from an undergraduate student to several individuals with more than 20 years of experience Other important identities shared by participants included status as a parent, first generation college attendee, or immigrants from countries outside the United States. Most geoscientists worked in academia, but not exclusively in faculty roles; all but one of the non-geoscience specific participants from focus groups conducted at braoder STEM-themed conferences were students early in their studies.Several participants had studied and worked in different institutions and settings and drew on their varied experiences to provide comparative commentary and examples. The data presented in the next section of this article are drawn primary from the geoscience conference focus groups, but our analyses and understanding were informed by the contributions of all participants.

## Findings and discussion

By drawing on first-person accounts shared during focus groups to illustrate themes and draw connections to the extant literature we provide an overall explanatory model for gender-based harassment and discrimination in the geosciences. The use of our conceptual framework, which links intersectionality and disciplinary norms in STEM fields, helped us to understand how our data set relates to broader phenomena and identify features unique to the geosciences. This lens also highlights the need to consider potential participants who had already been excluded or pushed out of the field, and whose voices were *not* reflected in the focus groups. All references to social identities included in this section use terms indicated by participants.

In order to illustrate the particular nature of these experiences in the discipline, we first present an overview of the types of incidents described to us during the focus groups, and then explain two primary drivers for this phenomenon: 1) a particular history of power dynamics and maintenance of dominant stereotypes, and 2) a pattern of ineffective responses to incidents of harassment and discrimination. direct quotations from participants are indicated in the text with italics but identifying details have been left out to ensure confidentiality.

### Experiences of harassment: Variations on a theme

As a phenomenon, harassment is frequently individualized, illustrated with descriptions of the experiences of particular survivors and characterized as exceptional. From an intersectional perspective, however, harassment is best understood as a structural problem and a reflection of accepted norms of behavior that position people with certain identities at particular risk; as noted by [68], "ignoring the structural arrangements of power leads to assumptions of equity [and] an overemphasis on the role of individual agency." During the focus groups, many participants shared specific examples of ways they had experienced or witnessed harassment and discrimination over the course of their careers. Although the persistence and frequency of these occurrences is already well-documented by many scholars [6–8, 47], we continue to encounter individuals who believe these problems occur in other STEM fields but are not convinced of their relevance to the geosciences. Rather than understanding power as constructing circumstances in which fairness is assumed despite unjust distributions of resources and opportunity, members of the community who individualize problems of bias and discrimination perpetuate an existing culture that tolerates abuse and harassment [67]. Here we seek not to sensationalize these reports, but rather to reiterate that these first-person accounts provide evidence that this is indeed an ongoing problem in the field—they are examples, not exceptions. As one advocate described in a 2020 conversation, "there’s a narrow line between respecting people’s survivor stories and convincing other people that this is a genuine issue;" here we aim to honor narratives while protecting privacy.

Psychologists frequently use a tri-partite model to explain sexual harassment, comprised of sexual coercion, unwanted sexual attention, and gender harassment [79]; these behaviors take place in a broader social context characterized by other forms of harassment and discrimination based on other identity markers [1]. Participants reported experiences of all three forms of harassment, with examples of gender harassment shared most frequently (echoing findings from other studies, e.g., [31]. While the examples of physical assault and clear sexual aggression we heard would likely be recognized as egregious by most people, many of the other examples presented by our participants were more subtle or allowed to persist for such lengths of time that they could be seen as routine elements of workplaces. The acceptance of such behavior disproportionately impacts those with multiple minoritized identities [67, 68]. Therefore, the impact is significant beyond the tolerance of sexual harassment in workplaces; intersectionality highlights oversimplified understandings of sexual and gender harassment as only reflections of sexism—rather than confluence of other normative forms of marginalization—are unlikely to lead to successful interventions [64]. Many participants noted how inappropriate language veiled as "humor" was common to their experiences in the geosciences; as one participant described, *it’s often dirty jokes that get laughed at the first time*, *and then it creates this environment of ’it’s okay to use that type of language*, *it’s okay to talk that way*. Another woman reported observing male colleagues treat other women (e.g., wait staff) in an objectifying manner, although similar behavior was not directed at her personally.

Participants also reported several examples in which women were afforded fewer opportunities for professional advancement despite equal qualifications with men. The explanations provided were also indicative of what has been [80] called a "Catch-22" for women: those seen as nurturing (especially those who were mothers) were seen as less serious about science, while those who were not viewed as caretakers were accused of being overly opinionated or shrill. One woman described how she was offered fewer research opportunities after becoming a parent early in her career because her supervisor assumed she would prefer to stay at home; almost two decades later, another woman recounted her story of being invited to babysit for the lab PI while a male counterpart was invited to co-author a feature paper. This example emphasizes how assumptions about gender have become an expected aspect of the geosciences, and therefore goes beyond only impacting women in the field. These built-in biases create expectations for behavior and gender for all members of the community.

Several participants reported experiencing more blatant harassment or disrespect at professional meetings away from their everyday workplaces. In a study of one professional society by [81], women reported experiencing more incidents of incivility, perceiving conferences as more sexist, and feeling more excluded than men. Often gender harassment occurs without a sexual advance [31]; many women reported receiving unsolicited "suggestions" about how they should adjust their physical presentation to be taken more seriously as scientists. Many participants reported receiving such inappropriate comments during poster sessions, when they were expecting to discuss results of research but would instead have to navigate critiques of their appearance and clothing. After explaining her research findings, one woman received the comment *you should dye your hair*, *you’re way smarter than you look*. This comment made her feel invalidated as a scientist, and also reflected assumptions about gender presentation rooted in sexist stereotypes that are often reinforced in male-dominated spaces [1]. These experiences can negatively impact scientists’ capacity to focus on their professional work—one woman described the stress imposed by attending a meeting in the presence of her harasser as *I can’t give a talk when I’ve just seen this guy walk in—it does slow down your mental processing*. The additional cognitive load imposed by navigating such conditions can reduce the attention available for a scientist to focus on work [57]. The data from our study demonstrate how sexual and gender-based harassment and discrimination are serious problems in the geosciences, as in other STEM fields.

### Power dynamics in the geosciences

Many aspects of work in STEM fields can reinforce hierarchies and imbalances of power [1]. Although harassment occurs across professional fields, our findings suggest that particular aspects of the geosciences as a broadly defined area of work and study create conditions under which unique forms of aggressive or exclusionary behavior can occur. Persistent stereotypes of individual geoscientists as white, male, and non-disabled is one factor that contributes to an "othering" of all women, People of Color, and disabled individuals that can lead to isolation, creating the conditions for harassment and discrimination [45, 46]. In one focus group, a stereotypical geoscientist was described as an *upper middle class White guy with a beard and a flannel shirt*; another participant noted fundamental ableism associated with an emphasis on field work: *the practice of geology as it currently exists*…*is limited to people who can physically undergo the training required*…*But thinking-wise*, *mind-capacity wise*, *I don’t think it inherently needs to be that way*. Other researchers have documented perceptions about accessibility barriers in the geosciences [82, 83] and illustrated how ableist assumptions marginalize disabled geoscientists. Because of the overall lack of representation in the geosciences, these stereotypes begin early in researchers’ careers, reflecting and reinforcing more widespread oppressive social forces. By shaping the contexts in which geoscientists develop personal understandings of the role of individual and collective behaviors in the field, these stereotypes become normative and can perpetuate hostile and unwelcoming climates [47]. In discussing what could help improve conditions, one participant’s response reflected the interconnectedness of forms of social oppressions, and they described the importance of incorporating anti-racist pedagogical approaches in coursework: *we should talk about a more diverse group of people and we should do those activities in a way that they’ll see themselves reflected with the students*, *like the student can see themselves in us and who we’re asking them to study so then they’ll feel like this is an area they can see themselves go into*. Relatedly, a woman of color geoscientist in a different focus group described the impact of never seeing anyone who looked like her throughout her undergraduate training as *I’ve never felt so excluded*. As described in a New York Times Opinion piece, "Earth Science has a whiteness problem" [84]—this piece was inspired by commentary [85] that called for geoscientists to interrogate how racialized assumptions and practices are deeply rooted in the field, and to work for systemic change in response. As conceptualized by Crenshaw, intersectionality calls upon researchers and advocates to avoid homogenizing group identities, and rather pay attention to intragroup difference in order to fully understand how racism, sexism, classism, and other forces of domination cannot be confronted in isolation [63].

Additional stereotyping of scientists as "objective" or assumptions of science as a socially neutral endeavor also contribute to the broader problem, particularly as forms of expression associated with femininity—especially outward displays of emotion—are seen as contrary to a professional geoscience approach. Previous research has documented how women in STEM fields often enter their studies intending to pursue careers that can accommodate personal life goals as well, and are often frustrated when they encounter inflexible work conditions [14]. Participants in our study shared examples of being told *there’s no crying in science* and being encouraged to compartmentalize their personal and professional identities. For individuals whose visible identities already highlight them as *different*, this request is impossible, further reinforcing how they are outside the norm and perpetuated through regular microaggressive acts and statements in the workplace. Intersectionality emphasizes that such responses are due not to the individual differences of specific geoscientists, but rather due to social power which creates marginalization and exclusion [63]. When one participant raised concerns about her treatment by colleagues, she was told she shouldn’t be *so sensitive*, and other women had similarly experienced being told they were *too emotional*, described as *overenthusiastic*, or criticized for being *too intense*. The continued dominance of masculinized expectations in terms of appearance and communication creates consequences for feminized expression—as one participant put it, *I’m essentially too female*. These norms do not only impact women, but rather constrain possibilities of gender identity and expression for all geoscientists and can have particularly negative consequences for LGBTQ+ individuals [22, 86]. These stereotypes reinforce the idea that in general, men and masculinity have greater power in the geosciences.

Early experiences in geoscientists’ professional development also impact their future career choices. As one participant described, being the target of bullying and incivility in the workplace made them feel…*like you don’t belong there*, *and you think maybe this is just not the place for me*. Another participant described how experiencing homophobic harassment from peers that was not disrupted by faculty supervisors early in his training impacted his trajectory; after receiving a message that he needed to *not act gay in the field* he decided to become a lab-based scientist to avoid future fieldwork. The impossibility of separating core personal identities from who they bring to work was described by this participant as *it’s not like you’re a scientist at work and then you’re queer at home*. Similarly, a woman who had initially hoped to conduct research in Antarctica was told early in her training that *there are no facilities for women* at the field station, so she could not go. These examples illustrate how most harassment in the workplace reflect hostility or antagonism against women and femininity, rather than outright sexual objectification [31, 56, 58]. Rather than targeting individuals, they function to exclude people with certain traits and maintain the status quo.

Rigid hierarchies associated with academic and research institutions and professional structures also reinforce existing mindsets and practices in the geosciences, especially assumptions about how science "should" be done and by whom. The competitive nature of applying for research grants advantages more senior scholars and those at more elite institutions, contributing to the elevation of certain researchers to "celebrity" status. The dependence of institutions on these researchers can also create circumstances in which they are allowed to engage in harassment and discrimination with impunity; a participant described how *one of our faculty who’s very distinguished [and] brings in a lot of money and has big grants made some very flagrant [racist] comments in a class setting*. Despite complaints by students and colleagues, this geoscientist faced no repercussions for his statements.

In one focus group, participants contrasted what they saw as a focus on research-intensive university work with the reality of most members of the community: *the assumption [that] all faculty are either tenured or tenure-track today*…*only about one in four has a tenure track position and everyone else is contingent or adjunct faculty—that has created a whole second tier that is the majority*. *They do most of the teaching*, *particularly in the large undergraduate courses*, *and they are mostly ineligible to submit [grant] proposals*. In many STEM fields there is more gender and racial diversity among graduate students and early career researchers [87]; therefore, many scientists from minoritized backgrounds are more likely to be in less well-paid or temporary positions [88]. This type of precarity contributes to climates that tolerate harassment and discrimination, as vulnerable employees feel more pressure to keep quiet about incidents they have experienced or witnessed, and to maintain favor on the part of supervisors and colleagues who may represent needed connections to funding or resources. Some women who participated in our focus groups and had achieved more "senior scholar" status expressed having more of an ability to speak up—as one put it, *I think maybe as I got older it was more okay to have an opinion*. Others expressed relief that with age they experienced less direct harassment, but due to a sense that older women become less visible to harassers (who move on to target younger scientists), not because of having earned respect: *as a ’grey hair’ I get accosted less physically which is lovely—I get ignored more*. When a certain degree of recognition is necessary for career advancement, a desire for invisibility can diminish scientists’ potential and productivity [89]. Alternatively, serial harassers who continue to receive funding and recognition as outstanding scientists become insulated from the impacts of their behavior, and it is targets that suffer the consequences. One participant described such a situation in which a tenured and high-profile scientist was allowed to remain in his position without sanction despite multiple reports of misconduct: *They knew that he’d done these horrible things*…*like they really*, *really dropped the ball on this one*. This lack of consequences [90] also reinforces existing power dynamics, further making it difficult for newer members of the community whose identities are "other" to gain status or advance in the field, and discourages those with minoritized identities, especially women of color, to envision themselves in the geosciences [2, 3, 26, 47].

### Ineffective responses to harassment and discrimination

Acknowledging the impact of hierarchy and existing structures is essential to understanding how existing systems ostensibly designed to respond to incidents of harassment and discrimination fail to adequately address these broader problems. Developed within and for institutions designed for white, abled, men from a narrow socioeconomic background, these systems tend to protect the existing power structure and place additional burdens on individuals outside dominant groups [1, 71]. In one focus group an early career scientist of color recounted an instance where they were the recipient of a racist statement in a public forum, and when no one spoke up in their defense they left the room. The individual reported: *I didn’t know what I could do*…*in the end I think things were said to this person*, *but nothing happened because they’re distinguished*. When established scientists are tacitly allowed to make racist comments in public with no penalty, other efforts to address diversity and inclusion are effectively undermined or made ineffective. Intersectionality highlights how racism as an oppressive social force is inherently connected to other issues such as sexism and anti-immigrant sentiment, for example [63]; the acceptance of one form of harassment or discrimination therefore creates the conditions for others.

Issues of confidentiality in reporting and response procedures were brought up in numerous focus group conversations as an additional stressor that prevented some from reporting incidents to begin with, or that weakened the efficacy of any response. Some reporting structures follow a mediation-style format, in which targets of harassment or discrimination are required to confront or explain themselves in a space where the perpetrator is also present and provided space to respond. In some circumstances, the person in the power position to whom incidents should be reported is themselves a harasser, or aligned with harassers, and not perceived as trustworthy—one faculty member reported working with a colleague who had targeted women in the department for years, but was protected by a close relationship with the college dean. One current undergraduate student described reporting incidents involving a particular faculty member to three separate people and experiencing three different responses, none of which were effective in preventing additional harassment; as they put it, *I just haven’t felt support from people in power*. Weak accountability mechanisms and lack of transparency on the part of leaders tasked with addressing harassment contribute to the prevalence of such problems [1].

Fear of retaliation was noted by many focus group participants as a deterrent to reporting behaviors; this echoes research that has documented the realities of such concerns [37,58, 90]. Other participants shared experiences that confirm the negative consequences that can come from ineffective or unsafe reporting mechanisms. In one example, a scientist reported a peer for sexual harassment, and in retaliation the harasser (who had seniority in the department) undermined her reputation to the graduate students working in her lab and even hired away her assistants. Another woman reported an assault perpetrated by a faculty colleague and found herself having to move offices and adjust her schedule to avoid contact with him, while no public response was made to address the behavior. In reflecting on her experience, she expressed a sentiment echoed by many participants—that perhaps they had not done something correctly: *I feel like in retrospect I should have called the police and done all these other things*, *and I was really worried about jeopardizing my job or my contract*. The failure in this situation is most certainly not on the part of the person assaulted, but often internalized as such; the hegemonic nature of widespread harassment can lead to internalization of responsibility rather than demanding accountability on the part of institutions [e.g. 58, 90]. Eventually she left the institution and took another position, while he remained. Such a response is common because institutional practices tend to encourage individualization of problems rather than recognizing incidents as indicative of broader cultures and structures. Legal and policy responses are often lacking because they originate from a social structure based on histories of discriminatory practice; ahistorical assumptions of meritocracy reinforce rather than counteract these underpinnings.

Several participants noted that responses that are in place on university campuses tend to prioritize student need while those of faculty or staff can be overlooked. One university scientist with student advising responsibilities reported feeling helpless to support a staff colleague who was being sexually harassed despite feeling empowered to advocate for students. Another participant noted how expectations were more ambiguous for those in non-student roles: *I would say at the student level there are resources that we have*. *You know you can tell someone about something that happened*…*But as a faculty or staff member I feel like you don’t know who you can really go to*, *or you can go to someone but then that trust is not there*. A similar sentiment was expressed by another woman: *Title IX [reporting] is better for the students*, *but as a faculty member I went to the EEO office and I left in tears—I felt as if THEY were harassing me*. Supporting this participant’s perceptions, research on Title IX implementation and enforcement documents that faculty experiences of harassment are underreported and frequently kept out of view [37].

The need to address power dynamics in a way that takes into account the broader field of the geosciences was referenced by several participants, who noted that although having advocates within their specific unit or workplace was important, having external reporting mechanisms was also essential. Scientists earlier in their careers want to know not only that they can find supportive mentors and advisors during their training and preparation, but that they will be able to move between institutions and research spaces throughout their careers and expect all workplaces to be free of harassment and discrimination. Further action or inaction at the leadership level is often thought to reflect the institution’s intentions [91]. Thus, institutional commitments that are transparent and consistently communicated by leadership [59] are therefore more significant for long-term protection than expressions of personal concern on the part of individuals.

### Significance and implications

The conceptual framework used in this study shows how power operates and perpetuates marginalization in the geosciences, and the excerpts from participants illustrate the specifics of this phenomenon. Our findings support the need for cultural and structural changes in the geosciences to combat gendered harassment and discrimination and to address the ways these phenomena are tied to race, class, sexual orientation, and ability. Given long-standing traditions and the continued dominance of identity norms in the field, these changes require the development and implementation of new practices that can transcend specific institutions [8, 23]. The efforts of the American Geophysical Union (AGU) to include exclusionary and harassing behaviors as scientific misconduct, for example, are an important step toward achieving these goals. AGU’s guidelines for scientific integrity and professional ethics also outline procedures for reporting and investigation of misconduct, including a range of enforceable sanctions, and have established new practices to provide support to attendees at in-person meetings [92]. The new policy adopted in 2017 extends beyond events organized by the association, and is being used to guide expectations for respectful conduct in academic departments and field research and training settings The new AGU Ethics and Equity center provides free legal consultation to early career society members regarding violations of the new code of conduct [93], which is one way to acknowledge the role of power dynamics in addressing these problems. The Paleontological Society in 2019 also adopted a new non-discriminatory policy and code of conduct for members in all professional and educational settings. The Geological Society of America (GSA) established a new program in 2018 with the hire of an ethics and compliance officer and new policies for reporting harassment, as well as a newly published members’ code of ethics in 2019 [94]. Additionally, GSA, like AGU, has made efforts to be transparent to their membership on how they are implementing the strategies identified in their new ethics framework. In 2020, GSA published an ethics report that outlined the number and types of complaints and reports received over a 3 year period [95]. This report also outlined the disciplinary steps that had been taken to address the complaints. Such moves acknowledge that these experiences impact scientific integrity as well as scientific productivity in the short-term and can restrict opportunities to continue in the field or achieve career advancement in the long-term. Government funding organizations, in particular the National Science Foundation and the National Institutes of Health, have also implemented new policies in the last couple of years to curtail research funding to investigated harassers. These actions by professional societies and funding agencies were taken in response to continued tolerance of harassment in academic environments [96] yet often still rely on flawed institutional processes for reporting and investigation [97].

The experiences reported in our data support critiques of existing harassment prevention mechanisms as limited and primarily effective in protecting institutions and documenting compliance, rather than encouraging cultural change. Although legal obligations can be important for the establishment of baseline expectations [37, 96], local policies and practices in particular workplaces are influenced by other norms and expectations. This is important to keep in mind when imagining the potential impact of professional society and funding agency changes. Intersectionality applied as a framework demonstrates the additional complexities faced by individuals who are members of more than one marginalized group because of overlapping oppressive forces in society; similarly, efforts to change the geosciences require an awareness that the history of social change movements does not document steady progress toward inclusion and awareness but rather cyclical struggles for recognition and redistribution of resources that challenge existing power structures [98].

The importance of leadership from individuals with positional authority was also identified as a crucial component of impacting change in local institutional settings and workplaces. Several participants noted how people in roles such as department chair or PI can use power in a positive manner—as one scientist described, *you’re department chair [and now] you can start saying stuff*, *[and] then other people can recognize that and that’s how you get the culture change*. In another focus group two women’s experiences highlighted the importance of field camp leadership. One described how *the head of the camp was an extremely bad example* during a season she described as overall unprofessional and probably dangerous, while the other participant shared a story of a highly functional and respectful field camp where safety was prioritized and productivity was high. [99] documented how fieldwork experiences were strongly influenced by the presence or absence of clear codes of conduct and the role of leadership. Leadership from geoscience-specific professional societies can also be key in setting the tone for broader cultural shifts that can support the work of change agents locally. Recent efforts to highlight the exclusionary nature of field work and field camp experiences to the Black, Indigenous, People of Color (BIPOC) and LGBTQ+ communities [25, 100–103] in addition to actions like that of AGU to classify harassment and discrimination as scientific misconduct, can be especially important in raising awareness that exclusionary and harassing behaviors in one location have consequences in others throughout the discipline. The NSF-funded ADVANCEGeo Partnership provides bystander intervention training for scientists at different career stages that employs discipline-specific scenarios, including fieldwork, and also offers workshops for writing effective codes of conduct, including for field research and training environments [61]. By adopting an intersectional framework, such as that described in this paper, ADVANCEGeo’s work responds to unique challenges of fieldwork, recognizing relationships between inclusivity, accessibility, and safety. GSA adopted new field safety policies and procedures in 2020 [104], and has started providing bystander intervention trainings to leadership and staff. AGU has been partnering with other organizations, including the American Geosciences Institute, to host webinars and trainings on implementing effective field safety policies [105].

Issues of individual representation continue to be very important in the geosciences because of persistent trends in low diversity. In our focus groups, for example, participants who were people of color were more likely to name racism as a factor that impacted the field overall; white people need to be more actively involved in naming and confront racial discrimination in their workplaces so that the onus is not placed on individual people of color [102]. A number of initiatives, many of them led by early career scientists in the geosciences, have brought attention to the need for community-wide engagement in improving diversity, equity and inclusion in the discipline by addressing workplace climate issues, recruitment, hiring, and retention, with an attention to social justice and recognition of the value of diverse epistemologies to the geosciences [98, 102, 106–108]. The coordinated efforts of historically excluded scientists need the support of more senior scientists in the field who are committed to changing, rather than replicating, existing structures. More work needs to be done on increasing representation in the geosciences beyond a focus on gender alone, which has been the main focus of past institutional efforts in STEM [109]. Some examples of relevant efforts include a widely circulated petition and article in *Nature* calling for a comprehensive plan for anti-racist efforts by geoscience organizations [110, 111], the formation of study and policy change study groups as part of the Unlearning Racism in Geoscience (URGE) initiative [112], coordinated activities on social media to promote BIPOC geoscientists such as #BlackinGeoscience [113], and the formation of grassroots networks such as GeoLatinas [114]. Persistent low racial and ethnic diversity in the geosciences, despite efforts to increase recruitment, highlight how the prevalent "leaky pipeline" model for overcoming inequities in STEM will not be successful unless attention is also focused on workplace behaviors such as harassment and racism that affect retention [115].

Finally, our analysis reflects how Black feminist scholarship provides a valuable framework for identifying the ways that addressing the underrepresentation of women in STEM without attending to the intersectional impacts of racism, colonialism, ableism, and classism will fail to diversify the geosciences [115]. Continuing to recruit individuals into a discipline without changing its fundamental nature can tokenize and isolate them or encourage assimilation and acceptance of deep-seated traditions no matter how damaging. It is the responsibility of those in power, and especially those who hold more privileged status due to their social identities, to contribute to the dismantling of current structures that reinforce inequity.

### Future research and action

In the immediate future, more substantive action is needed to stop existing practices that only superficially address incidences of harassment and discrimination but allow perpetrators of these behaviors to remain in the geosciences. This includes increased sanctions such as the removal of funding or awards, and positions of power that provide undue influence over the career paths of others, like graduate students and postdoctoral researchers. We collectively need to support an expansion of research that documents and collects evidence about practices in the geoscience that are effective in shifting the focus of early career development away from hyper-individualistic capacity and status-building to one that more holistically supports science as a collective enterprise. Scientists who acknowledge the historical and political contexts in which their work occurs more fully and accurately position their own work in a complex social context [116] and can create space for new innovation and discovery [117]. It is our hope that this article is one step toward revealing how long-established and accepted practices and behaviors in the geosciences have contributed to lack of diversity and exclusion in the field—but also to suggest ways that change for the future is possible.

## Supporting information

S1 Appendix (DOCX)
