# Supplementary material for: "Maybe this is just not the place for me:" Gender harassment and discrimination in the geosciences
Source: PLoS One. 2022 May 18;17(5):e0268562. doi: 10.1371/journal.pone.0268562 (PMC9116675; doi:10.1371/journal.pone.0268562)
Supplement: S1 Appendix — (DOCX) [file pone.0268562.s001.docx]

Appendix A: Semi-Structured Focus Group Protocol

1. Please introduce yourself and let us know what identities you bring to your work as a scientist (what aspects of your identity are most salient for you in your workplace?) or describe what is relevant to your identity as a scientist. For example, … [[*facilitator introduces themselves briefly*]]

1. Do you think there is a characteristic culture or set of social norms typical of your discipline or sub-field? (clarification/probing questions: Is there a way geoscientists, for example, are expected to be, act, or think? **or** For example, did anybody warn you about the culture of your department or field?)

1. This project is focusing on empowering communities to address the problem of sexual harassment, What are some interventions you have witnessed on campus, in the field, or the workplace that effectively disrupt sexual harassment? You may have seen an effective disruption of another type of harassment that you think may also work towards sexual harassment.

1. We want to develop intervention strategies and training materials that serve diverse communities. How do you think sexual orientation, gender identity and expression, and/or racial and ethnic or Native identity affect the way that the scientists in your discipline are perceived or treated by their peers?

1. What kinds of training have you received about preventing sexual harassment on campuses or in the workplace as a student or employee? Did you think this training was effective? Why or why not? What additional training or interventions do you think would help improve conditions for scientists?
